# The clinical features and prognostic factors of miliary tuberculosis in a high tuberculosis burden area

**DOI:** 10.1080/07853890.2024.2356647

**Published:** 2024-06-07

**Authors:** Xiaolin Wei, Min Xie, Suji Wu, Yong Bao

**Affiliations:** a Department of Respiratory, Sichuan Taikang Hospital, Chengdu, Sichuan, P. R. China; b Department of Pulmonary and Critical Care Medicine, West China Hospital, Sichuan University, Chengdu, Sichuan, P. R. China

**Keywords:** Miliary tuberculosis, clinical features, prognostic factors

## Abstract

**Background:**

Miliary Tuberculosis (TB) remains an important infectious disease that threatens human health. The clinical characteristics and prognostic factors of miliary TB are summarized in this study.

**Methods:**

The clinical information of miliary TB patients between 2010 and 2022 was retrospectively analyzed. Patients with miliary TB were characterized and compared to adverse outcomes cases. Factors independently associated with adverse outcomes were determined via multivariate logistic regression analysis.

**Results:**

A total of 288 patients were analyzed, including 181 with adverse outcomes. The clinical manifestations are atypical. 88.54% Of them experienced systemic symptoms, whilst 69.79% manifested respiratory symptoms. 40.97% Presented with neurologic symptoms, while 35.07% reported gastrointestinal symptoms. The major comorbidities were pharmacological immunosuppression (21.53%), pneumoconiosis (15.28%), diabetes (10.76%), and pregnancy or postpartum (7.29%). Regarding microbiology, most patients were diagnosed via sputum or Bronchoalveolar Lavage Fluid (BALF), pleural effusion, ascites, cerebrospinal fluid, urine TB-DNA, and tuberculosis culture. Meanwhile, 2.43% of patients were diagnosed via cerebrospinal fluid NGS. Independent risk factors predictive of adverse outcomes were current smoking, leukocytosis, elevated alanine aminotransferase (ALT) levels, and the combination of lymphopenia with bone marrow tuberculosis or tuberculous lymphadenitis. The accuracy of the model was validated by an area under the ROC curve of 0.753 (95% IC 0.697–0.810).

**Conclusions:**

The clinical manifestations of miliary TB are atypical, and early diagnosis is challenging. The major comorbidities in miliary TB patients were pharmacological immunosuppression, pneumoconiosis, diabetes, pregnancy, and postpartum. Regarding etiological detection, multi-site and multi-type specimens should be collected for a timely diagnosis. Cerebrospinal fluid mNGS test may be a viable choice in some cases. Finally, current smoking, leukocytosis, elevated ALT levels, and the combination of lymphopenia with bone marrow tuberculosis or tuberculous lymphadenitis were identified as independent risk factors for adverse outcomes.

## KEY MESSAGE

The clinical manifestations of miliary TB are atypical, and early diagnosis is challenging. The major comorbidities in miliary TB patients were pharmacological immunosuppression, pneumoconiosis, diabetes, pregnancy, and postpartum. Current smoking, leukocytosis, elevated ALT levels, and the combination of lymphopenia with bone marrow tuberculosis or tuberculous lymphadenitis were identified as independent risk factors for adverse outcomes.

## Background

Tuberculosis (TB) remains an important infectious disease that threatens human health, a major cause of poor health, and the leading cause of death from a single infectious agent [1–3]. In 2021, an estimated 10.6 million people were newly infected with TB worldwide, along with an estimated 1.6 million TB deaths (including 187,000 individuals living with HIV) [2]. Previous studies have established that one-third of the global population is infected with *Mycobacterium tuberculosis* (latent TB infection), with 10% of these individuals progressing to active TB in their lifetime [4]. The COVID-19 pandemic has also negatively impacted tuberculosis diagnosis and treatment, thereby elevating the burden of the disease [2,5].

Miliary tuberculosis is a fatal disseminated TB caused by a large number of Mycobacterium tuberculosis through lymphatic or hematogenous dissemination [6]. Despite its relatively low incidence, the diverse clinical manifestations and atypical imaging features of miliary TB frequently lead to delayed diagnosis and high mortality rates [7]. Previously, miliary TB was considered a disease of infants and children; however, with the HIV/AIDS epidemic increasing the use of immunosuppressants and biologics, it has become increasingly prevalent in adults as well [6,8]. In many cases, early, effective anti-tuberculosis treatment can enhance disease prognosis. Nevertheless, severe complications and adverse drug reactions, combined with extrapulmonary tuberculosis, immunosuppressive hosts, and resistance to first-line anti-tuberculosis drugs may yield poor clinica outcomes [8,9]. Consequently, screening and monitoring for complications such as acute respiratory distress syndrome (ARDS), adverse drug reaction, and comorbidities are warranted. However, the sample size of the majority of existing cohorts is small, with limited imaging and microbiological data [10–13].

Therefore, it is essential to identify the clinical features and critical risk factors associated with adverse outcomes in miliary TB patients, given that increased awareness may result in better clinical outcomes. Our research aimed to investigate clinical features and identify predictors of adverse outcomes associated with miliary TB. In addition, the clinical course, findings, and adverse outcomes of a cohort with miliary TB from a high TB-burden country were characterized, which may assist in formulating effective preventive and intervention strategies.

## Methods

### Study design

A retrospective analysis of miliary tuberculosis patients admitted to West China Hospital of Sichuan University from 2010 to 2022 was performed, focusing on clinical characteristics and laboratory test results. The university-affiliated hospital located in Sichuan, West China, an area with a high incidence of tuberculosis (TB). Patients aged <14 years or those with missing information (medical history, examination, and imaging) were excluded. Ethics approval was granted by the Ethics Board of the Institute of West China Hospital of Sichuan University (Ethics Board of West China Hospital of Sichuan University, 2022-1366).

### Data analysis

Data on epidemiology, demography, clinical characteristics, laboratory tests, treatment, and outcomes were acquired from the electronic medical records of West China Hospital and reviewed by two infectious disease doctors. Disagreements were resolved and adjudicated by a third researcher.

### Definition

Subjects were categorized according to the disease site, with miliary TB defined as the pathological manifestation of millet seed-sized granulomas in lungs affected by tubercle bacilli. Miliary infiltrates on chest HRCT were classified as typical (multiple 1–2 mm well-defined nodules throughout the lungs) or atypical (predominant nodules that measured >2 mm) miliary pattern [14]. The diagnosis of miliary TB was established based on the presence of clinical and radiological signs and confirmed by aetiological diagnosis, pathological diagnosis, or therapeutic response [15]. The diagnosis of acute respiratory distress syndrome (ARDS) was based on the Berlin definition of ARDS [16]. Adverse outcomes were defined as the need for invasive mechanical ventilation, development of ARDS, ICU admission, death during hospitalization, treatment discontinuation, and length of stay >14 days.

### Statistical analysis

Statistical analyses were performed using R (version 4.0.5). Normally distributed data were expressed as mean ± standard deviation. For numerical data, the *T*-test was used for the comparison of two groups of means, whereas ANOVA was used for the comparison of multiple groups of means. The Chi-square test or Fisher exact test was utilized to compare classified data. Univariate analyses were conducted to compare features of general miliary TB and miliary TB with adverse outcomes. Multivariable logistic regression was used to identify independent predictors for adverse outcomes. All variables associated with adverse outcomes with a *p* < 0.20 in the univariate analysis were introduced in the multivariate model. During model construction, variables that were not significant or those with collinearity were excluded. *p* < 0.05 Was considered statistically significant. The results of significant predictors were reported as odds ratios (OR) and 95% confidence intervals (CI). The accuracy of the model was evaluated using the receiving operator characteristic (ROC) analysis.

## Results

### Sample characterization

A total of 313 cases of miliary pulmonary tuberculosis were diagnosed in hospitalized patients at West China Hospital of Sichuan University from 2010 to 2022. Among them, 25 patients aged <14 years or with incomplete information were excluded, whilst 288 patients were retained. Multiple hospitalizations for the same reason were only included in the initial analysis. Finally, the data of 107 general patients and 181 patients with adverse outcomes were examined. The baseline clinical characteristics of the patients are presented in Table 1. There were fewer women in the study population (*n* = 131). Han, Tibetan, Yi, and other ethnic minorities accounted for 71.53% (*n* = 206), 18.40% (*n* = 53), 8.33% (*n* = 24), and 1.74% (*n* = 5) of the cohort, respectively. There was no significant difference in the source, drinking habits, and department distribution between the two groups. Hospitalized patients with miliary pulmonary tuberculosis were largely distributed in the tuberculosis department, respiratory department, and infection department. Regarding clinical presentation, 88.54% (*n* = 255) of patients manifested systemic symptoms such as fatigue, anorexia, and fever. 69.79% (*n* = 201) of patients presented with respiratory symptoms such as cough, sputum, and dyspnea, while 40.97% (*n* = 118) developed headache, dizziness, and disturbance of consciousness, and 35.07% (*n* = 101) patients experienced gastrointestinal symptoms such as nausea and vomiting. Regarding comorbidities, the top four most common comorbidities in miliary TB patients were pharmacological immunosuppression (21.53%), pneumoconiosis (15.28%), diabetes (10.76%), and pregnancy or postpartum (7.29%). Concerning microbiology, the majority of patients were diagnosed with tuberculosis through sputum or Bronchoalveolar Lavage Fluid (BALF). Specifically, 10.07% (*n* = 29) of patients were diagnosed via sputum or BALF acid-fast staining, 38.19% (*n* = 110) via sputum or BALF TB-DNA, and 14.93% (*n* = 43) through sputum or BALF tuberculosis culture. At the same time, a proportion of patients tested positive in samples collected from pleural effusion, ascites, cerebrospinal fluid, urine TB-DNA, and blood. Histopathological examination displayed granuloma, caseous necrosis, positive acid-fast staining, and tuberculosis PCR positive in 24.31% (*n* = 70) of patients. 7.64% (*n* = 22) of patients were diagnosed by Next Generation sequencing (NGS), among which 2.43% (*n* = 7) tested positive using samples collected from cerebrospinal fluid.

**Table 1.** Clinical characteristics of miliary tuberculosis.

Characteristics	All, *N*=, *n*(%), mean (SD)	No poor outcomes, *N* = 107	Poor outcomes, *N* = 181	*p* Value
Age, mean (SD)	42.6 ± 8.59	41.79 ± 18.74	43.17 ± 18.54	0.543
Female gender, *n* (%)	131 (45.49)	41 (38.32)	90 (49.72)	0.079
Ethnic, *n* (%)				0.063
Han	206 (71.53)	71 (66.36)	135 (74.59)	
Tibetan	53 (18.40)	19 (17.76)	34 (18.78)	
Yi	24 (8.33)	15 (14.02)	9 (4.97)	
Others	5 (1.74)	2 (1.87)	3 (1.66)	
Marital status, *n* (%)				0.564
Unmarried	74 (25.69)	30 (28.04)	44 (24.31)	
Married	202 (70.14)	74 (69.16)	128 (70.72)	
Divorced/widowed	12 (4.17)	3 (2.80)	9 (4.97)	
Source, *n* (%)				0.164
Urban	116 (40.28)	37 (34.58)	79 (43.65)	
Rural	172 (59.72)	70 (65.42)	102 (56.35)	
Previous history of TB, *n* (%)	21 (7.29)	9 (8.41)	12 (6.63)	0.743
Smoking habits, *n* (%)				0.032
Non smoker	230 (79.86)	77 (71.96)	153 (84.53)	
Former smoker	33 (11.46)	18 (16.82)	15 (8.29)	
Smoker	25 (8.68)	12 (11.21)	13 (7.18)	
Drinking habits, *n* (%)				0.543
Non drinker	243 (84.38)	87 (81.31)	156 (86.19)	
Former drinker	16 (5.56)	7 (6.54)	9 (4.97)	
Drinker	29 (10.07)	13 (12.15)	16 (8.84)	
Time of symptoms, days, mean (SD)	3.72 ± 8.46	3.39 ± 4.45	3.92 ± 10.11	0.612
Length of stay, days, mean (SD)	20.28 ± 13.45	9.98 ± 2.98	26.36 ± 13.53	<0.001
Department distribution, *n* (%)				0.308
Tuberculosis	194 (67.36)	79 (73.83)	115 (63.54)	
Respiratory	41 (14.24)	15 (14.02)	26 (14.36)	
Infection	14 (4.86)	5 (4.67)	9 (4.97)	
Neurology	9 (3.12)	3 (2.80)	6 (3.31)	
Rheumatology and immunology	4 (1.39)	1 (0.93)	3 (1.66)	
Nephrology	4 (1.39)	0 (0.00)	4 (2.21)	
Others	22 (7.64)	4 (3.74)	18 (9.94)	
Symptoms, *n* (%)				
Respiratory system	201 (69.79)	76 (71.03)	125 (69.06)	0.827
Digestive system	101 (35.07)	34 (31.78)	67 (37.02)	0.440
Nervous system	118 (40.97)	39 (36.45)	79 (43.65)	0.282
Urinary system	5 (1.74)	0 (0.00)	5 (2.76)	0.205
Constitutional symptoms	255 (88.54)	95 (88.79)	160 (88.40)	1
Others	38 (13.19)	15 (14.02)	23 (12.71)	0.891
Comorbidities, *n* (%)				
Diabetes	31 (10.76)	9 (8.41)	22 (12.15)	0.427
Chronic kidney disease	14 (4.86)	7 (6.54)	7 (3.87)	0.462
Organ transplantation	4 (1.39)	1 (0.93)	3 (1.66)	1
Pharmacological immunosuppression	62 (21.53)	17 (15.89)	45 (24.86)	0.101
Active cancer	10 (3.47)	3 (2.80)	7 (3.87)	0.886
HIV	12 (4.17)	7 (6.54)	5 (2.76)	0.213
Malnutrition	5 (1.74)	2 (1.87)	3 (1.66)	1
Chronic liver failure/cirrhosis	13 (4.51)	3 (2.80)	10 (5.52)	0.435
Pregnancy or postpartum	21 (7.29)	7 (6.54)	14 (7.73)	0.887
Pneumoconiosis	44 (15.28)	20 (18.69)	24 (13.26)	0.285
Others	16 (5.56)	3 (2.80)	13 (7.18)	0.193
System involvement, *n* (%)				
Secondary pulmonary TB	75(26.04)	29 (27.10)	46 (25.41)	0.86
Tuberculous pleurisy	47(16.32)	21 (19.63)	26 (14.36)	0.316
Nervous system involvement	119(41.32)	40 (37.38)	79 (43.65)	0.358
Bone marrow tuberculosis	32(11.11)	3 (2.80)	29 (16.02)	0.001
Tuberculosis of genitourinary system	23(7.99)	8 (7.48)	15 (8.29)	0.98
Tuberculous pericarditis	17(5.9)	6 (5.61)	11 (6.08)	1
Bone tuberculosis	26(9.03)	11 (10.28)	15 (8.29)	0.721
Abdominal and pelvic involvement	72(25)	24 (22.43)	48 (26.52)	0.526
Lymph node involvement	31(10.76)	17 (15.89)	14 (7.73)	0.05
Other extrapulmonary TB	8(2.78)	2 (1.87)	6 (3.31)	0.726
Microbiology, *n* (%)				
Acid-fast staining positive, *n* (%)				
Sputum or BALF	29 (10.07)	12 (11.21)	17 (9.39)	0.769
Other specimen	6 (2.08)	2 (1.87)	4 (2.21)	1
TB-DNA positive, *n* (%)				
Sputum or BALF	110 (38.19)	43 (40.19)	67 (37.02)	0.682
Pleural fluid	5 (1.74)	1 (0.93)	4 (2.21)	0.738
Ascites	5 (1.74)	2 (1.87)	3 (1.66)	1
Urine	12 (4.17)	2 (1.87)	10 (5.52)	0.232
Cerebrospinal fluid	35 (12.15)	7 (6.54)	28 (15.47)	0.04
Other specimen	11 (3.82)	3 (2.80)	8 (4.42)	0.709
Culture-positive, *n* (%)				
Sputum or BALF	43 (14.93)	19 (17.76)	24 (13.26)	0.388
Cerebrospinal fluid	11 (3.82)	4 (3.74)	7 (3.87)	1
Urine	3 (1.04)	1 (0.93)	2 (1.10)	1
Ascites	4 (1.39)	3 (2.80)	1 (0.55)	0.291
Others	2 (0.69)	1 (0.93)	1 (0.55)	0.769
Histopathology supports tuberculosis, *n* (%)	70 (24.31)	26 (24.30)	44 (24.31)	1
mNGS positive, *n* (%)	22 (7.64)	7 (6.54)	15 (8.29)	0.757
TB antibody positive, *n* (%)	167 (57.99)	59 (55.14)	108 (59.67)	0.529
Tuberculin test positive (PPD test), *n* (%)	25 (8.68)	15 (14.02)	10 (5.52)	0.024
Interferon-gamma release test positive, *n* (%)	193 (67.01)	76 (71.03)	117 (64.64)	0.325
Therapy, *n* (%)				
Hormone	141 (48.96)	40 (37.38)	101 (55.80)	0.004
Antituberculosis regimen, *n* (%)	181 (62.85)			
First-line treatment	82 (28.47)	39 (36.45)	43 (23.76)	0.03
Second-line treatment (quinolones combined with first-line drugs)	154 (53.47)	54 (50.47)	100 (55.25)	0.507
Other second-line regimen	85 (29.51)	27 (25.23)	58 (32.04)	0.275
Adverse outcomes, *n* (%)				
Invasive mechanical ventilation	18 (6.25)	0 (0.00)	18 (9.94)	0.002
Development of ARDS	31 (10.76)	0 (0.00)	31 (17.13)	<0.001
ICU admission	21 (7.29)	0 (0.00)	21 (11.60)	0.001
Treatment discontinuation	17 (5.90)	0 (0.00)	17 (9.39)	0.003
Death during hospitalization	3 (1.04)	0 (0.00)	3 (1.66)	0.46
Length of stay >14 days	161 (55.90)	0 (0.00)	161 (88.95)	<0.001

*Definition of abbreviations*: TB: tuberculosis; HIV: human immunodeficiency virus; BALF: bronchoalveolar lavage fluid; mNGS: metagenomic next generation sequencing; ARDS: acute respiratory distress syndrome; ICU: intensive care unit.

### Laboratory tests

Leukocytosis, neutrophilia, lymphopenia, hypoalbuminemia, and elevated levels of direct bilirubin, alanine aminotransferase (ALT), aspartate aminotransferase (AST), lactic dehydrogenase (LDH), and serum creatinine (Scr) were more commonly observed in the poor outcome group. Likewise, CRP levels and erythrocyte sedimentation (ESR) rates were higher in the adverse outcome group, although the differences were not statistically significant. In contrast, leukopenia and elevated total bilirubin levels were more prevalent in the general group. The details are listed in Table 2.

**Table 2.** Initial laboratory analysis.

Initial laboratory analysis	All, *N*=, *n* (%), mean (SD)	No poor outcomes, *N* = 107	Poor outcomes, *N* = 181	*p* Value
Complete blood count				
White blood cell count; 10^9^/L; normal range 3.5–9.5				
Increase	40 (13.89)	7 (6.54)	33 (18.23)	0.009
Decrease	49 (17.01)	22 (20.56)	27 (14.92)	0.285
Neutrophil count; 10^9^/L; normal range 1.8–6.3				
Increase	21 (7.29)	2 (1.87)	19 (10.50)	0.013
Lymphocyte count; 10^9^/L; normal range >1.1				
Decrease	222 (77.08)	73 (68.22)	149 (82.32)	0.009
Haemoglobin; g/L; normal range >130				
Decrease	226 (78.47)	78 (72.90)	148 (81.77)	0.105
Platelet count; 10^9^/L; normal range 100–300				
Decrease	66 (22.92)	32 (29.91)	34 (18.78)	0.043
C-Reactive protein; mg/L; normal range <10				
Increase	233 (80.90)	85 (79.44)	148 (81.77)	0.741
ESR; mm/h; normal range <15				
Increase	247 (85.76)	91 (85.05)	156 (86.19)	0.926
Blood biochemical analysis				
Total bilirubin				
Increase	26 (9.03)	11 (10.28)	15 (8.29)	0.721
Direct bilirubin				
Increase	57 (19.79)	14 (13.08)	43 (23.76)	0.041
ALT; U/L; normal range <50				
Increase	65 (22.57)	14 (13.08)	51 (28.18)	0.005
AST; U/L; normal range <40				
Increase	109 (37.85)	29 (27.10)	80 (44.20)	0.006
Serum creatinine; Scr; mmol/L; normal range <111				
Increase	28 (9.72)	9 (8.41)	19 (10.50)	0.71
Plasma albumin; g/L; normal range >35				
Decrease	175 (60.76)	61 (57.01)	114 (62.98)	0.38
LDH; U/L; normal range <250				
Increase	135 (46.88)	47 (43.93)	88 (48.62)	0.516

*Definition of abbreviations*: ESR: erythrocyte sedimentation rate; ALT: alanine aminotransferase; AST: aspartate minotransferase; Scr: serum creatinine; LDH: lactic dehydrogenase.

### Imaging examinations

Among the whole cohort, 86.81% (*n* = 250) exhibited typical miliary nodules, 23.61% (*n* = 68) had atypical nodules, 34.38% (*n* = 99) demonstrated lymph node involvement, and 32.64% (*n* = 94) presented with a patchy shadow. As anticipated, pleural involvement was observed in a considerable number of patients, with pleural effusion (28.13%, *n* = 81) or pleural thickening (29.17%, *n* = 84) being more common. In addition, pericardial effusion was noted in some patients (22.22%, *n* = 64). The details are summarized in Table 3.

**Table 3.** Imaging examinations (%).

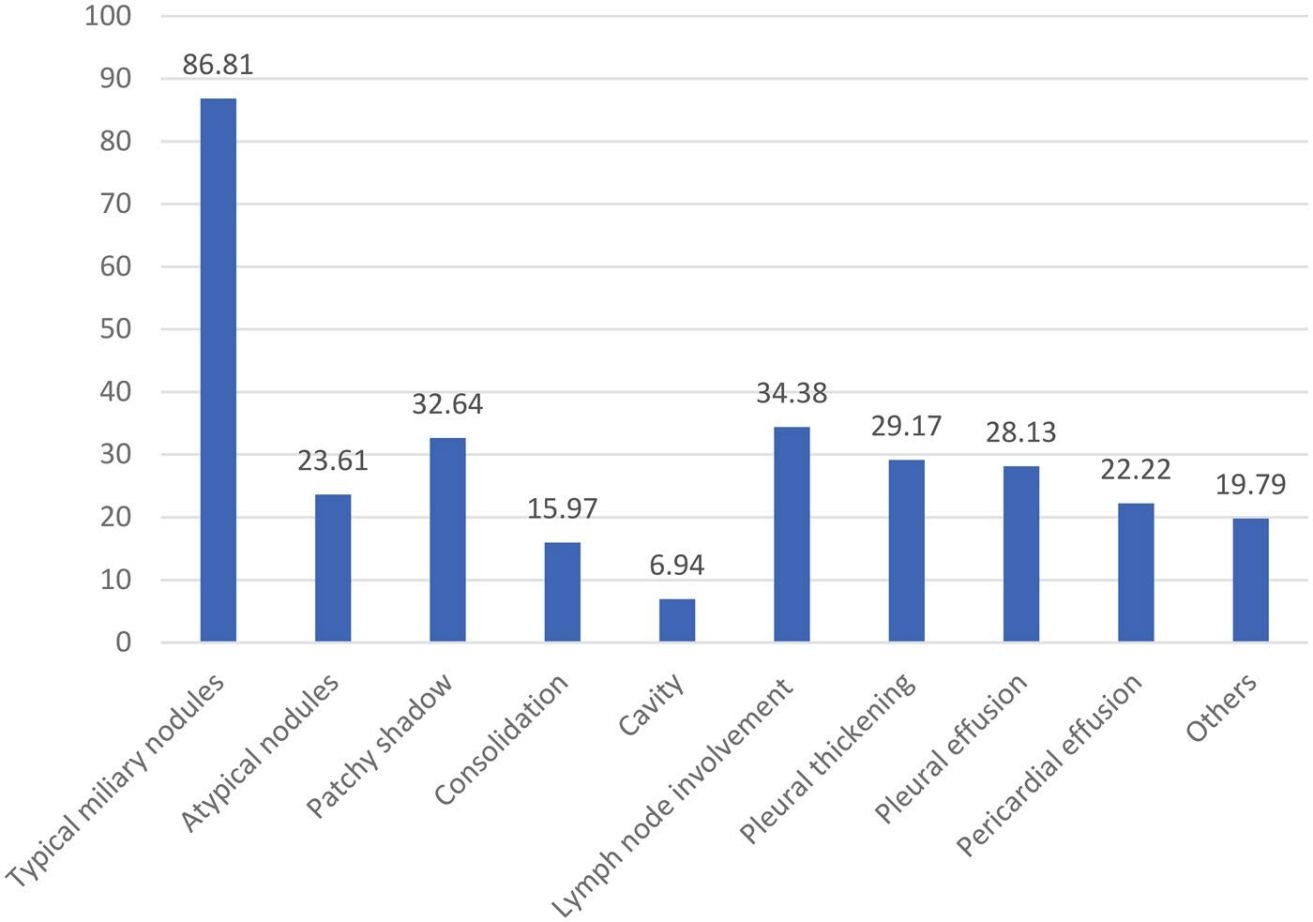

### Common symptoms

77.08% (*n* = 222) Of patients developed fever in the whole cohort, with more than half developing a cough (54.51%, *n* = 157). Meanwhile, night sweats were reported by nearly one-fifth of patients (19.79%, *n* = 57), and a relatively small number of patients experienced hemoptysis (1.39%, *n* = 4). See Table 4.

**Table 4.** Common symptoms(%).

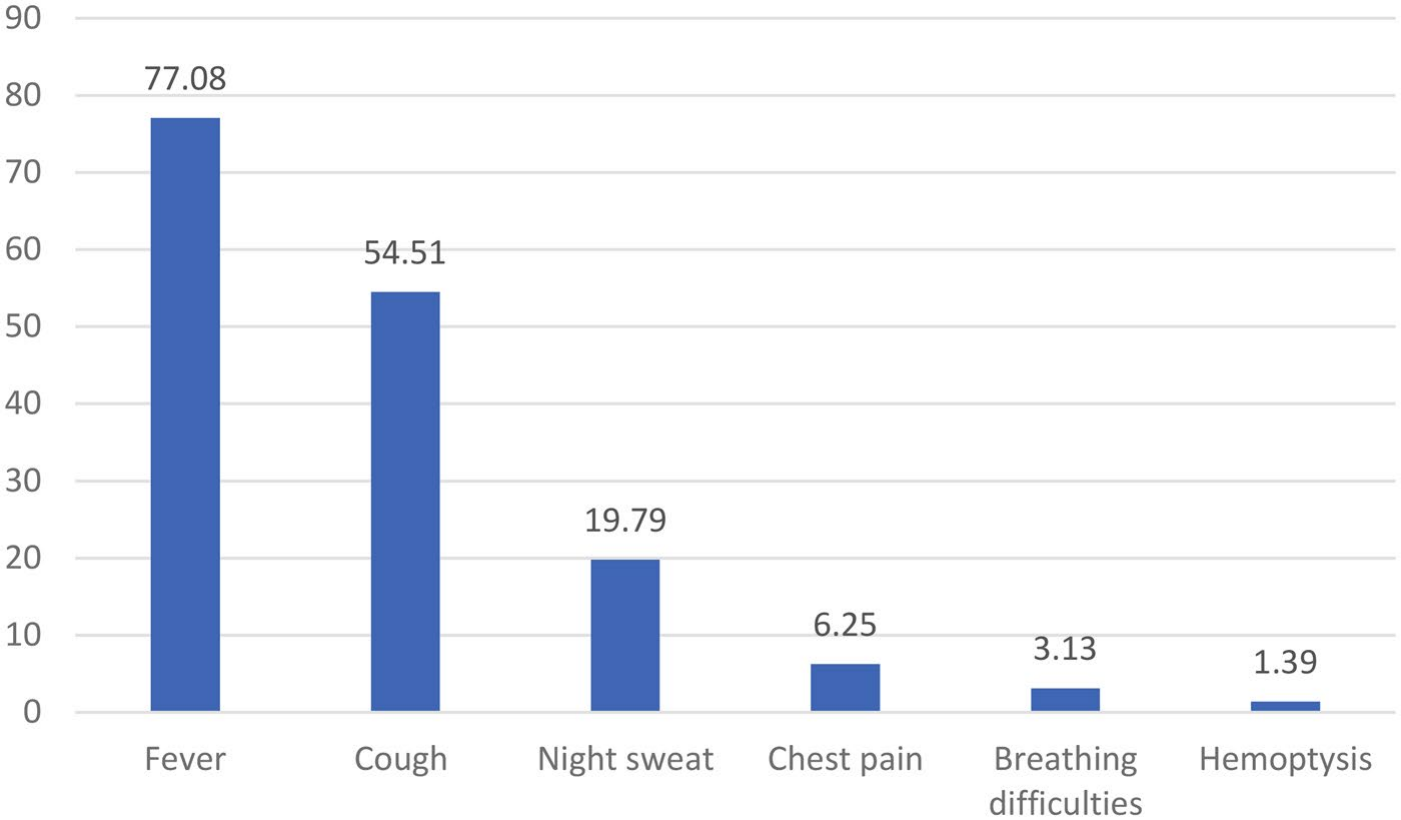

### Systemic disseminated tuberculosis

Regarding systemic disseminated tuberculosis, up to 41.32% (*n* = 119) of patients had central nervous system involvement, 26.04% (*n* = 75) had secondary pulmonary tuberculosis, and 25% (*n* = 72) had abdominal and pelvic involvement. Importantly, 16.32% (*n* = 47) and 11.11% (*n* = 32) of patients developed tuberculous pleurisy and bone marrow tuberculosis, respectively. In addition, lymph node tuberculosis (10.76%, *n* = 31) and bone tuberculosis (9.03%, *n* = 26) were observed in some patients (Table 5).

**Table 5.** Combined with systemic disseminated tuberculosis (%).

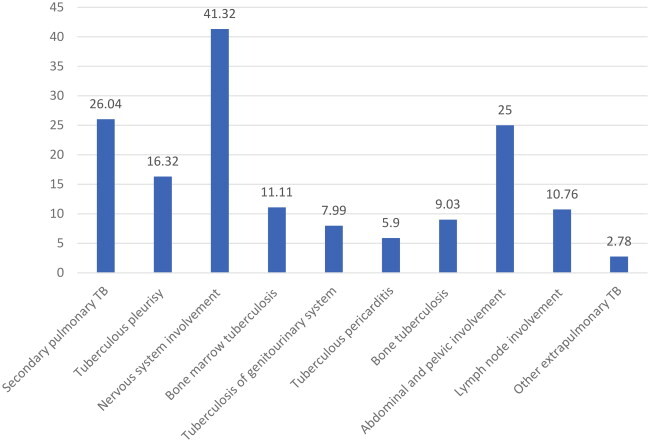

### Treatment and outcomes

The adverse outcome group was more likely to be given second-line regimens, including quinolones and other second-line antituberculosis therapy, and also more likely to receive hormones, which was in line with our clinical experience. In the study cohort, a total of 181 (62.83%) experienced adverse outcomes. As detailed in Table 1, 6.25% (*n* = 18), 10.76% (*n* = 31), 7.29% (*n* = 21), 5.90% (*n* = 17), 1.04% (*n* = 3), and 55.90% (*n* = 161) of patients received invasive mechanical ventilation, developed ARDS, underwent ICU admission, discontinued treatment, experienced death during hospitalization, and had a length of stay >14 days, respectively.

### Identification of risk factors for adverse outcomes

A multivariate logistic regression model was developed to identify risk factors for adverse outcomes. Patients were stratified by age, gender, nationality, marital status, and so on. The following continuous variables were transformed into binary factors using specific thresholds: White blood cell count <3.5 × 10^9^/L or >9.5 × 10^9^/L, Neutrophil count >6.3 × 10^9^/L, Lymphocyte count <1.1 × 10^9^/L, hemoglobin level <13 g/dL, Platelet count <100 × 10^9^/L, C-Reactive protein level >10 mg/L, ESR >15 mm/h, Total bilirubin level >28 μmmol/L, direct bilirubin level >8.8 μmmol/L, ALT level >50 U/L; AST level >40 U/L, Serum creatinine level >111 μmol/L, albumin level <35 g/L, LDH level >250 U/L.

**Figure 1.**
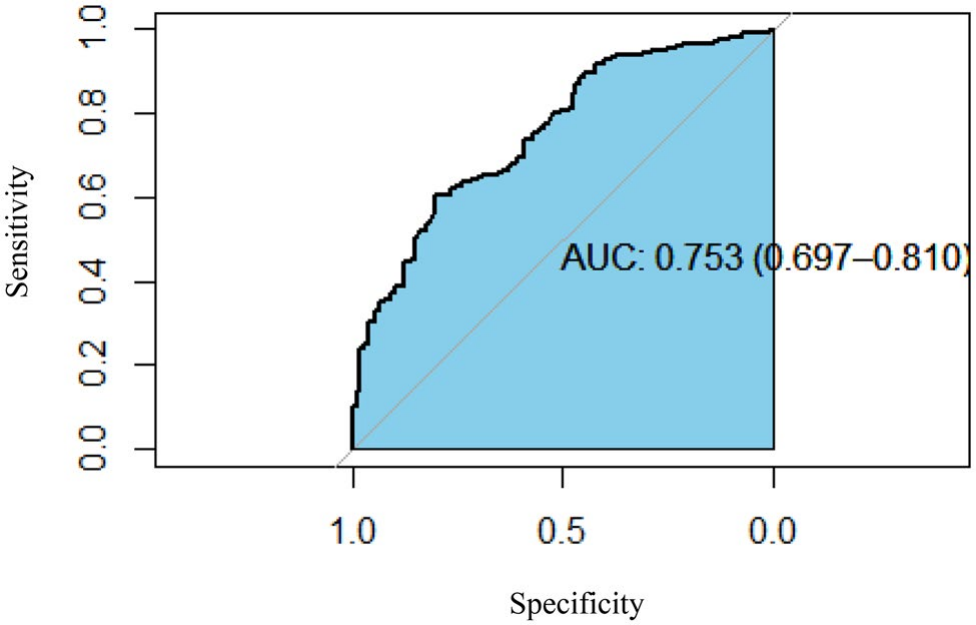
The receiving operator characteristic (ROC) analysis of independent predictors for adverse outcomes.

As listed in Table 6, independent risk factors for adverse outcomes were identified as follows: current smoking, leukocytosis, elevated ALT levels, and lymphopenia combined with bone marrow tuberculosis or tuberculous lymphadenitis. The accuracy of the model was confirmed with an area under the ROC curve of 0.753 (95% IC 0.697–0.810), as illustrated in Figure 1.

**Table 6.** Multivariate analysis of adverse outcomes.

	Estimate	OR	95%CI	Std. error	*z* Value	Pr(>|*z*|)
Former smoker	−0.36	0.698	0.385 ∼ 1.247	0.298	−1.206	0.228
Smoker	−1.285	0.277	0.114 ∼ 0.639	0.435	−2.951	0.003
Neutrophilia	−0.696	0.499	0.195 ∼ 1.264	0.473	−1.472	0.141
Lymphopenia	1.726	5.618	1.399 ∼ 38.512	0.808	2.137	0.033
Elevation of AST	0.768	2.155	1.176 ∼ 3.987	0.311	2.473	0.013
Leukocytosis	0.689	1.992	1.13 ∼ 3.575	0.293	2.351	0.019
Bone marrow tuberculosis	1.554	4.730	1.476 ∼ 21.482	0.663	2.344	0.019
Lymph node involvement	−0.857	0.424	0.178 ∼ 0.98	0.432	−1.984	0.047
Other Comorbidities	1.174	3.235	0.93 ∼ 15.222	0.691	1.698	0.090
Pharmacological immunosuppression	0.585	1.795	0.935 ∼ 3.567	0.34	1.722	0.085

## Discussion

Among the 288 patients with miliary pulmonary tuberculosis, 181 patients experienced adverse outcomes. Age was comparable between the two groups. Interestingly, the incidence of miliary TB was higher in individuals with Tibetan and Yi nationalities compared with the Han ethnicity. This observation may be ascribed to the underdeveloped economy and poor sanitary conditions in Tibetan and Yi areas in western Sichuan. Nonetheless, the impact of ethnic variation or host genetic factors on the development of miliary TB warrants further investigation. Our study exposed that the most common comorbidities of miliary tuberculosis patients include pharmacological immunosuppression 21.53% (*n* = 62), pneumoconiosis 15.28% (*n* = 44), diabetes 10.76% (*n* = 31), pregnancy or postpartum 7.29% (*n* = 21), chronic kidney disease 4.86% (*n* = 14), chronic liver failure/cirrhosis 4.51% (*n* = 13), HIV/AIDS 4.17% (*n* = 12) and active cancer 3.47% (*n* = 10), which is in line with the findings of existing studies. Meira, L [17]. described that HIV infection, pharmacological immunosuppression, and diabetes are major risk factors for disseminated tuberculosis. Noteworthily, Kaige Wang et al. [18] evinced that pregnant women, especially women undergoing in-vitro fertilization (IVF), are more prone to developing miliary tuberculosis. This may be attributed to the use of glucocorticoids in IVF treatment and increased oestradiol secretion during pregnancy. Of note, both can suppress the immune system, making pregnant women vulnerable to TB infection or relapse. Similarly, Sharma S.K [6–8]. pointed out that pneumoconiosis, active cancer, and pharmacological immunosuppression are susceptible factors for patients with miliary tuberculosis.

The clinical manifestations of miliary tuberculosis are non-specific, and few typical imaging features appear in the early stage, thus making early diagnosis challenging [7]. Common clinical symptoms herein included fever, fatigue, cough, expectoration, and dyspnea. Given that miliary TB can target multiple organs, patients may present with symptoms and signs related to various organ systems, such as nausea, vomiting, abdominal pain, headache, and disturbance of consciousness, which are generally consistent with patients with miliary TB [6,8,18]. It is worthwhile emphasizing that fever may also lead to headaches. Common side effects of anti-tuberculosis drugs include gastrointestinal reactions such as nausea and vomiting [19]. The diagnosis of tuberculosis requires evidence from multiple aspects, including medical history, clinical manifestations, signs, imaging, microbiology, and histopathology. The diagnosis of miliary pulmonary tuberculosis primarily relies on chest imaging examination. Notably, chest HRCT has been established to outperform chest X-rays in terms of sensitivity. Typical HRCT findings of diffuse distribution of 1–2 mm typical miliary nodules in bilateral lungs are indicative of miliary pulmonary tuberculosis [6,14,20]. However, some patients with multiple atypical pulmonary nodules, especially those immunocompromised or with ARDS and mixed infections, may easily be misdiagnosed [6,13,21]. Herein, typical miliary nodules were identified in 86.81% of cases, in line with the findings of Kwong JS et al. [22], who detected nodules measuring greater than 3 mm in diameter in 10% of miliary TB cases. Meanwhile, 34.38% of the cases manifested lymph node enlargement, while a considerable number of patients presented with hydrothorax, seroperitoneum, and pericardial effusion, which is consistent with the study finding of Pipavath et al. [23]. Furthermore, numerous patients exhibited patchy consolidation, cavitation, calcification, fiber cords, and other image findings in the lungs, which reflected the polymorphism of tuberculosis imaging features. Moreover, it is pivotal to improve the diagnostic ability for atypical tuberculosis and identify lesions other than tuberculosis, such as fungal infection, malignant tumor, NTM, and sarcoidosis [21,24]. As is well documented, etiology remains the gold standard for the diagnosis of tuberculosis. Regrettably, the specificity of body fluid acid-fast staining smears is low, and mycobacterial culture is time-consuming, generally exceeding 2 weeks, with a specificity of merely 20–30% [25]. Likewise, cerebrospinal fluid (CSF) GeneXpert MTB/RIF has a low sensitivity [26]. In the present study, most cases were diagnosed by detecting tuberculosis in sputum or BALF, but a small number of patients were diagnosed via metagenomic next-generation sequencing (mNGS) of blood, biopsy, and cerebrospinal fluid specimens, with cerebrospinal fluid accounting for 2.43%. This signifies that cerebrospinal fluid mNGS plays a diagnostic role for miliary tuberculosis, especially in cases with atypical imaging and negative tuberculosis detection in respiratory specimens, in accordance with the study of Yuanting Ye et al. [27] and Sun, W. et al. [28].

Our study revealed that current smoking, leukocytosis, elevated ALT levels, and the combination of lymphopenia with bone marrow tuberculosis or tuberculous lymphadenitis were independent risk factors for adverse outcomes. Several earlier studies have also linked cigarette smoke exposure and tuberculosis infection, active tuberculosis, and tuberculosis-related mortality [29–31]. According to a prior investigation, the frequencies of both M1 and M2 macrophages and levels of MMP9 and MMP12 in bronchoalveolar lavage were increased in pulmonary TB patients who were smokers [29]. Additionally, another study showed that social habits such as alcohol consumption and active smoking could exacerbate the symptoms of TB [32]. Noteworthily, several studies demonstrated that leukocytosis was closely related to the prognosis of miliary TB [33,34]. A related study showed that patients with altered mental status, leucocytosis, and thrombocytopenia had a poor prognosis [33]. Furthermore, a study conducted in the Philippines determined that leukocytosis was significantly associated with mortality in genitourinary tuberculosis patients [34]. T lymphocytes play a crucial role in anti-TB immunity. Lymphopenia was found in nearly half of untreated pulmonary TB patients and three-quarters of patients with miliary TB [35,36]. Consequently, lymphopenia is associated with disease severity in patients with TB [37]. Underwood, J. et al. observed that admission ALT ≥180 IU/L was independently associated with the need for critical care intervention and mechanical ventilation [12]. Furthermore, Maartens et al. unconvered that lymphopenia and elevated transaminase levels were predictors of adverse outcomes in patients with miliary TB [38]. Systemic organ involvement, combined with extrapulmonary TB, usually leads to poorer outcomes compared to pulmonary TB only [39]. Studies identified a strong correlation between bone marrow tuberculosis or tuberculous lymphadenitis with adverse outcomes. The former is often associated with bone marrow suppression and severe cases can be associated with hemophagocytic syndrome (HPS) and the emergence of drug-resistant tuberculosis [40,41]. Besides, the lymphatic system is most frequently affected in extrapulmonary TB patients. Bilateral painless cervical lymphadenitis is the most frequent manifestation of lymph node involvement. Fever, weight loss, and weakness can also manifest, especially in HIV-positive patients [42]. A study related to tuberculous lymphadenitis unveiled that a considerable proportion of patients had comorbidities such as diabetes mellitus (DM), human immunodeficiency virus (HIV), and hepatitis [42].

Owing to the retrospective single-center nature of this study, we could not determine the prevalence of pulmonary tuberculosis or miliary tuberculosis in this area. Besides, our study, conducted in a large hospital, chiefly manages difficult and complicated diseases in West China. As a result, patients may be more critically ill, leading to the underrepresentation of less severe cases.

## Conclusions

The major comorbidities in miliary TB patients were pharmacological immunosuppression, pneumoconiosis, diabetes, pregnancy, or postpartum. The non-specificity of signs and symptoms may impede a timely diagnosis of miliary TB and contribute to this globally rare but severe disease. In terms of etiological detection, the use of multi-site and multi-type specimens is recommended to facilitate early diagnosis. Cerebrospinal fluid mNGS test may be the preferred choice in some cases. Finally, current smoking, leukocytosis, elevated ALT levels, and the combination of lymphopenia with bone marrow tuberculosis or tuberculous lymphadenitis were identified as independent risk factors for adverse outcomes.

## Data Availability

Access to data is regulated by Chinese law. Data are available from Sichuan University Hospital for researchers who meet the criteria required by Chinese law for access to confidential data. The contact person will distribute data upon request to qualified researchers: Suji Wu, Department of Pulmonary and Critical Care Medicine, West China Hospital, Sichuan University, 1457456392@ qq.com.
